# Congenital hepatoblastoma presenting with hepatic arteriovenous fistulas: a case report

**DOI:** 10.3389/fped.2023.1199224

**Published:** 2023-07-13

**Authors:** Jiao Lin, Jialing Guo, Chun Chen, Liqiong Jiang, Can Lai, Chunlin Wang

**Affiliations:** ^1^ Department of Pediatrics, The First Affiliated Hospital, Zhejiang University School of Medicine, Hangzhou, China; ^2^ Department of Radiology, Children's Hospital, Zhejiang University School of Medicine, National Clinical Research Center for Child Health, Hangzhou, China

**Keywords:** congenital hepatoblastoma, hepatic arteriovenous fistulas, metastasis, diagnosis, therapy

## Abstract

**Aim:**

Congenital hepatoblastoma, a rare malignant liver tumor in infancy, typically presents with abdominal distension or mass. Tumors detected antenatally or during the first three months of age are considered congenital hepatoblastoma. Hepatic arteriovenous fistulas (HAVF) are associated with high mortality in the neonatal period and can be caused by many secondary factors. This case report focuses on a patient with congenital hepatoblastoma accompanied by HAVF, highlighting the clinical and imaging characteristics and management strategies.

**Case presentation:**

A term infant presented with sudden tachypnea and heart failure on his first day of life. A cystic-solid mixed lesion in the fetus’s liver was detected by an antenatal ultrasound scan. Postnatal digital subtraction angiography confirmed the presence of arteriovenous fistulas, which were treated with trans-arterial embolization. However, despite the intervention, the patient’s heart failure did not improve. The patient underwent a left hepatectomy, and hepatoblastoma was discovered by histology of the resected hepatic lobe. Unfortunately, metastases were later discovered in the intracranial and ocular regions. Ultimately, the family decided to discontinue further treatment.

**Conclusion:**

Congenital hepatoblastoma presenting with hepatic arteriovenous fistulas has not been previously described. Hepatoblastoma should be considered when alpha-fetoprotein levels show a significant elevation in newborns. Prenatal diagnosis may improve pre- and postnatal management.

## 1. Background

Hepatoblastoma is a rare hepatic malignant tumor primarily affecting children under three years old. Congenital hepatoblastoma detected antenatally or during the first three months of age accounts for less than 10% of all pediatric hepatoblastomas. Congenital hepatoblastoma is associated with a worse prognosis, as it tends to have a higher incidence of pure fetal histology and a higher probability of metastasis (1). Hepatic arteriovenous fistulas (HAVF) are aberrant connections between the hepatic artery and portal or the hepatic vein. According to the etiology, HAVF can be divided into congenital and acquired. The latter is most common, with less than 10% being congenital (2). The clinical manifestations depend on the types of abnormal hepatic blood supply and the size of the shunts (3). The present article retrospectively analyzed the clinical data of a neonate with heart failure and liver mass, initially considered congenital HAVF, and finally diagnosed as hepatoblastoma.

## 2. Case report

**Figure 1:**
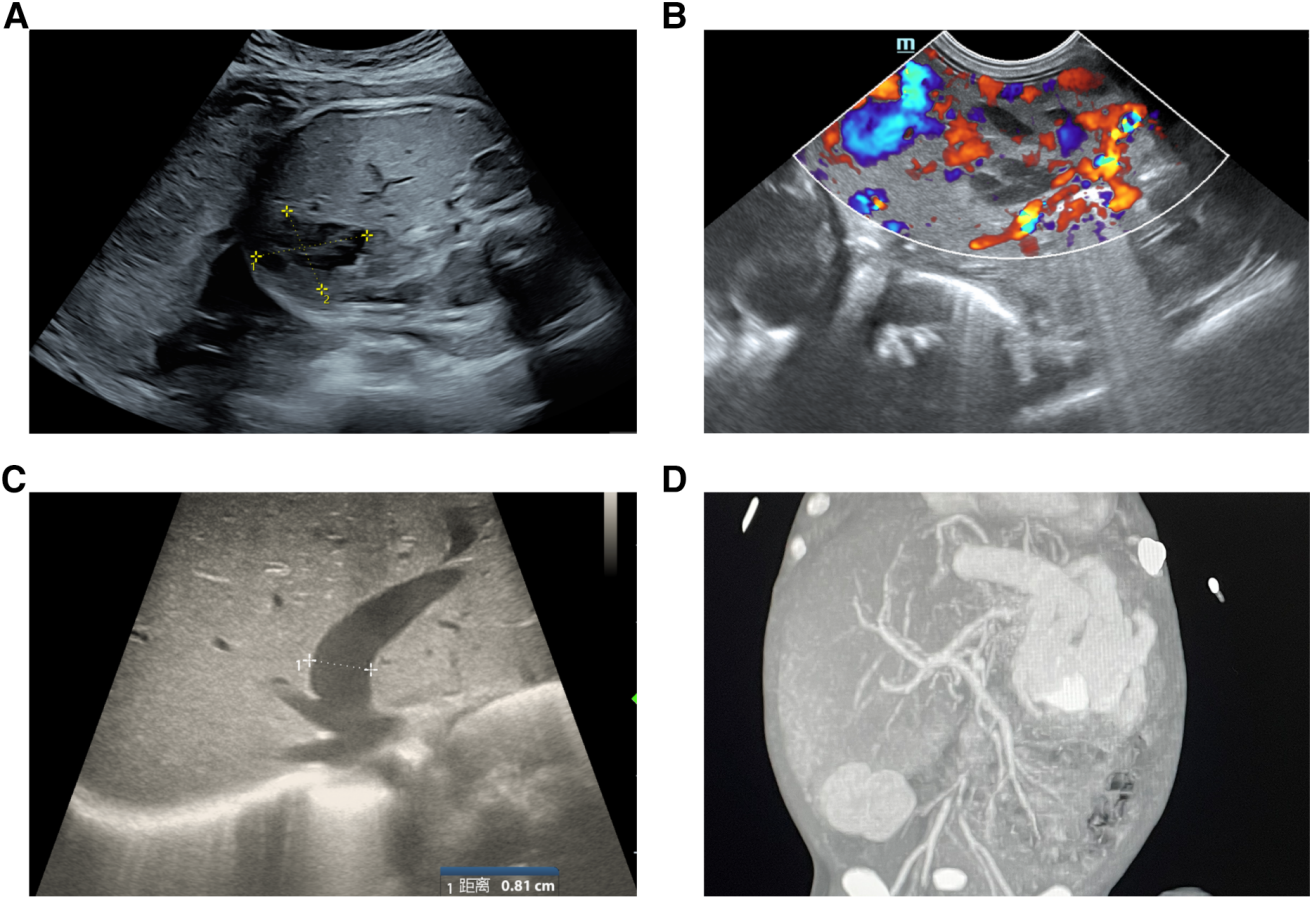
(**A**) Prenatal ultrasound findings at 28 weeks gestation showed a large, cystic and solid mass (3.6 × 3.6 × 3.0 cm) in the left lobe of the liver. (**B**) Postpartum hepatic ultrasonography showed a 5.3 × 4.7 × 3.3 cm mass with abundant blood signal. (**C**)The diameter of the left hepatic vein was dilated (0.8 cm). (**D**) Enhanced CT revealed a large arteriovenous fistula in the left hepatic lobe.

**Figure 2:**
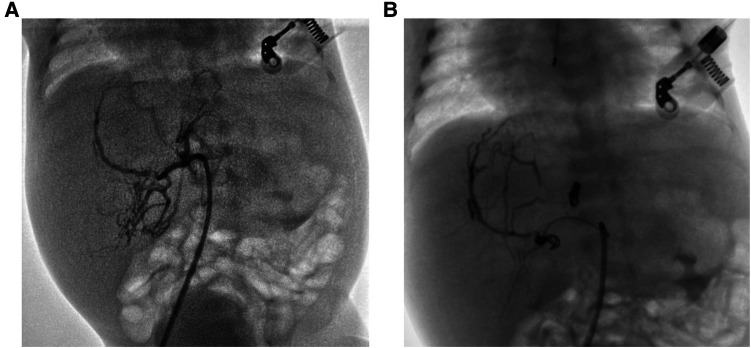
(**A**) The hepatic angiography showed arteriovenous fistulas. (**B**) The fistulas reduction after embolization.

**Figure 3:**
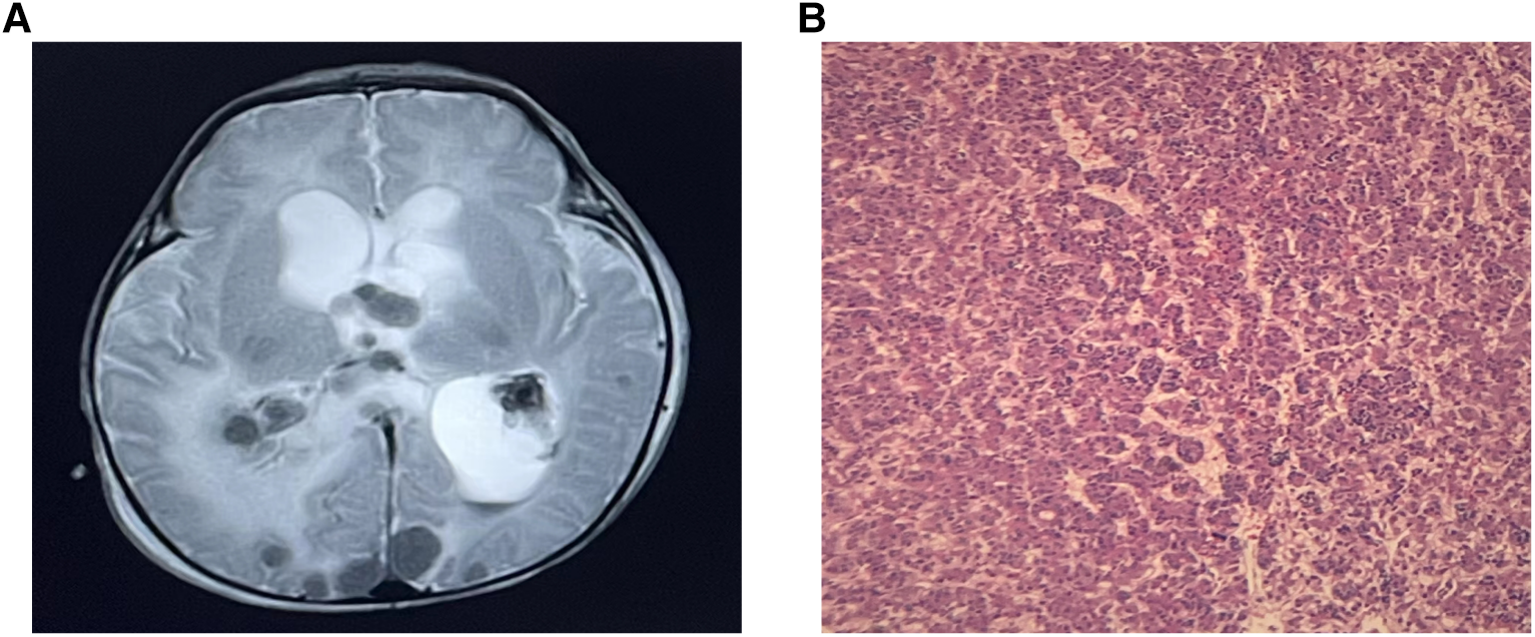
(**A**) Brain MRI demonstrated multiple intracranial tumors. (**B**) The pathology revealed an epithelial-type hepatoblastoma.

A male infant was admitted to NICU at 19 h of life due to jaundice and tachypnea. The boy was 38 + 2 weeks of gestation. The birth weight was 2,290 g (less than the 5th percentile), and the length was 44 cm. Apgar scores were 10 and 10 in the 1st and 5th minutes. The mother was 37 years old with antiphospholipid antibody syndrome and a history of retroperitoneal tumor resection. An ultrasound taken at 28 weeks of gestation revealed the hepatic space-occupying lesion to be 3.6 cm × 3.6 cm × 3.0 cm (Figure 1A). At 29 weeks, prenatal MRI revealed a globular mass located over the left lobe of the liver with hypointensity on T1W1, hyperintensity on T2W1, and hypointensity on DWI. The mass was diagnosed as hepatic hemangioendothelioma at that time. On physical examination upon admission, the boy presented with mild tachypnea. A grade 3/6 continuous murmur was auscultated in the upper left sternal border and a distended abdomen was observed with the liver palpated 3 cm below the costal margin. ABO incompatibility was confirmed through neonatal direct antiglobulin testing. The boy’s liver function tests were normal (AST 37 U/L, ALT 5 U/L), but his alpha-fetoprotein (AFP) level was relatively high (406,212 ng/ml). The brain natriuretic peptide (BNP) level was elevated to 2,254 pg/ml. Echocardiography revealed an atrial septal defect (3 mm) and pulmonary hypertension. Postpartum hepatic ultrasonographic examination showed a 5.3 cm × 4.7 cm × 3.3 cm mass with abundant blood signal (Figure 1B). Furthermore, there was an increase in the diameter of both the left hepatic vein (0.8 cm) and the hepatic artery (0.4 cm) (Figure 1C). Contrast CT of the abdominal microvasculature demonstrated that the abnormal blood vessels originated from the hepatic artery and drained into the inferior vena cava through the branches of the middle and left hepatic veins (Figure 1D). On the 3rd day, the boy presented with respiratory distress and heart failure. To manage heart failure, we administered dobutamine, milrinone, and ventilator-assisted breathing. Considering the need for surgery and intervention, the patient was transferred to the local children’s hospital and underwent transcatheter embolization therapy. A sheath was placed into the right femoral artery, and a 5F catheter was placed into the abdominal aorta. Abdominal aortography demonstrated an artery-hepatic vein fistula supplied by the left hepatic artery and draining into the hepatic veins. Moreover, the right hepatic artery connecting with the portal vein formed the artery-portal vein fistula (Figure 2A). To treat the fistulas, we inserted three spring coils to embolize the hepatic artery branch (Figure 2B). However, the clinical condition did not improve after embolization. After discussion in a multidisciplinary session, a left hepatectomy was performed on the postnatal 20th day. On the 6th postoperative day, the patient exhibited symptoms of intracranial hypertension, and a brain MRI scan revealed multiple intracranial tumors (Figure 3A). Meanwhile, the pathology of the resected hepatic lobe revealed an epithelial-type hepatoblastoma (mixed fetal and embryonal) (Figure 3B). To relieve hydrocephalus, we performed a ventriculoperitoneal shunt, and the patient underwent C5VD chemotherapy (cisplatin, 1, 5-fluorouracil, vincristine, and doxorubicin). However, tumor metastasis was also detected in the eyes and right liver. Eventually, the family made the decision to discontinue further treatment.

## 3. Discussion

HAVF is a rare condition with a low incidence rate and is often misdiagnosed. It was first reported by Goodhart et al. in 1899 and is classified into hepatic artery-portal vein fistula (HAPVF) and hepatic artery-hepatic vein fistula (HAHVF) (4). The clinical presentation varies depending on the type and size of the fistula (5). The clinical symptoms of HAHVF include heart failure, arrhythmia, and pulmonary hypertension. The direct flow of hepatic artery blood into the hepatic vein via the fistula leads to increased precardiac load and subsequent cardiovascular manifestations (6). Contrarily, the signs of HAPVF are related to the progression of portal hypertension, such as hepatomegaly, splenomegaly, ascites, upper gastrointestinal bleeding, and chronic diarrhea. Small HAPVF can be asymptomatic (2).

In the present case, the infant rapidly presented with respiratory distress and heart failure. Considering the prenatal ultrasound findings, we suspected that an intrahepatic space-occupying lesion caused congestive cardiac failure. Meanwhile, enhanced CT and digital subtraction angiography (DSA) were performed to confirm the diagnosis. The current main treatment options for HAVF include interventional embolization, hepatic artery ligation, and hepatic lobectomy (7). Interventional embolization has the advantages of short hospitalization time and rapid recovery. Surgery is applied for complicated lesions or failure of the intervention. A recent study demonstrated that the beta blocker was effective. Hong (8) reported the successful use of a small dosage of propranolol (1 mg/kg/day) in two neonates with HAVF, resulting in a significant reduction of the fistula. However, the clinical condition of the patient did not improve. Consequently, a left hepatectomy was performed as a treatment measure. After surgery, the patient exhibited symptoms of intracranial hypertension. Whereas HAVF did not affect the brain, further examinations were performed, and the final postoperative pathology revealed the presence of hepatoblastoma. The discovery of hepatoblastoma elucidated the underlying cause of the patient’s symptoms and clinical manifestations.

Compared with HAVF, hepatoblastoma is more common in infant, however, it remains a rare disease with an incidence is 11.2 per million (9). Generally, imaging and AFP are crucial in diagnosis, while tissue biopsy remains the gold standard. Notably, more than 90% of cases have highly elevated serum levels of AFP (10), a valuable biomarker for evaluating treatment effectiveness and monitoring recurrence. Both low serum AFP (<100 ng/ml) and high AFP (>10,000,000 ng/ml) are poor prognostic factors (11). In the present case, the patient had risk factors, including low birth weight and a significant increase in AFP; however, no significant evidence of hepatoblastoma was found through multiple imaging examinations. Ultimately, the diagnosis was confirmed through pathology. Therefore, we should be aware of the possibility of liver tumors in newborns with significant AFP elevation. Congenital hepatoblastoma has the characteristics of early onset, rapid progression, easy metastasis, and compression effect (12). In our case, eye and brain metastases were found, and the family chose to abandon treatment. Although previous reports have clarified that the prognosis of congenital patients is not worse than that of older patients (9), the specific circumstances of this case may have contributed to unfavorable outcomes. Beate et al. evaluated 1,605 patients with hepatoblastoma and found that children younger than 28 days had a comparable prognosis to other children under 1 year of age at the time of diagnosis (13). However, the presence of multiple metastases was associated with a poor prognosis. Hu et al. retrospectively analyzed 36 hepatoblastoma patients with metastases between 2010 and 2017. All patients were treated with surgery and chemotherapy, with nine alive at the time of analysis (14). Parul revealed that only 4/13 patients with intracranial metastasis survived for six months (15). Even though congenital hepatoblastoma does not confer a worse prognosis, the early occurrence of metastasis should still be a concern. This finding emphasizes the need for further research on metastatic hepatoblastoma to enhance the survival rate and improve outcomes for affected individuals.

The relationship between the two diseases must be considered, and on the basis of monism, we hypothesized that hepatoblastoma led to the development of HAVF. First, HAVF is common in primary hepatocellular carcinoma with an advanced stage, frequently involving the hepatic venous and/or portal venous system (7). Hepatoblastoma is also a highly aggressive tumor that can cause vascular invasion (16). Second, there have been reports of hepatoblastoma occurring concurrently with congenital portosystemic shunts in several cases. Timothy B. described two children with a portosystemic shunt and a liver mass diagnosed as hepatoblastoma on surgical biopsy (17). It was speculated that the presence of a portosystemic shunt could lead to a disordered proliferation of hepatocytes, eventually resulting in tumor formation (18). However, the patient in our case had a liver mass in the perinatal period, which was inconsistent with long-term vascular malformation leading to a tumor. To the best of our knowledge, no such report has been documented for hepatoblastoma presenting with HAVF, and this may provide new insights into the clinical manifestations of hepatoblastoma.

With the increasing availability of prenatal examinations, prenatal diagnosis plays a crucial role in detecting fetal hepatic tumors and allows for appropriate perinatal care and definitive treatment. The evaluation methods for fetal hepatic tumors include fetal ultrasound and MRI. The characteristic sonographic findings of hepatoblastoma revealed a large, hypoechoic solid lesion in the liver with focal hemorrhage, necrosis, and calcification (1). MRI shows predominantly T2-hyperintense and T1-hypointense with areas of heterogeneity caused by hemorrhage or necrosis (19). Ultrasound findings during HAVF may reveal tortuous and enlarged blood vessels within a specific liver lobe, and multiple direct communications between the hepatic artery and vein branches. Spectral analysis revealed an increased in arterial and venous flow velocity. MRI can efficiently assess HAVF extent and size, with a decreased signal on Tl-weighted images and increased signal on T2-weighted images.

Although our patient had a poor outcome, we believed that early antenatal diagnosis of hepatoblastoma and HAVF in other cases could lead to more favorable outcomes. Prenatal diagnosis allows for timely intervention and appropriate management, ultimately improving the prognosis for affected infants.

## 4. Conclusion

We reported a case of a rare hepatoblastoma presenting with HAVF in an infant. The patient presented with heart failure and was ultimately diagnosed through DSA and histological examination. The present case highlighted the significance of radiological features and AFP levels in the early detection of hepatoblastoma. A timely diagnosis permits consideration of prompt treatment and may lead to better outcomes.

## Data Availability

The original contributions presented in the study are included in the article, further inquiries can be directed to the corresponding author.
